# The central role of peers facilitators in the empowerment of breast cancer patients: a qualitative study

**DOI:** 10.1186/s12905-022-01892-x

**Published:** 2022-07-24

**Authors:** Béatrice Lognos, Isabelle Boulze-launay, Million Élodie, Gérard Bourrel, Michel Amouyal, Xavier Gocko, Clary Bernard, Grégory Ninot, Agnès Oude Engberink

**Affiliations:** 1 University of Montpellier, Montpellier, France; 2 University of Montpellier, IDESP, 641 avenue du Doyen Gaston Giraud, 34090 Montpellier, France; 3 Université Paul-Valéry, Montpellier, France; 4 Jean Monnet University, Saint Etienne, France; 5 Department of General Practice, Faculté de Médecine Montpellier 641 Av. du Doyen Gaston Giraud, 34000 Montpellier, France; 6 MSPU, MSPU Pauline Lautaud, avenue d’Occitanie, 34680 ST Georges d’Orques, France; 7 MSPU, MSPU Cabestany, 2 Rue Ibn Sinaï dit Avicenne, 66330 Cabestany, France; 8 MSPU, MSPU La Source, 68 Rue du Charron, 30310 Vergèze, France; 9 MSP, MSP Le Caducée, 20 Rte Minervoise, 11800 Trèbes, France

**Keywords:** Empowerment, Peers, Patient association, Breast cancer, Phenomenological study

## Abstract

**Background:**

In 2020, the number of new cancer cases was estimated at 20 490 862 worldwide up from 18.1 million in 2018 and 14.1 million in 2012. Since the 2000s, cancer treatments have significantly improved, allowing either a cure or control of the disease. Patients share their experience of the disease and use supportive care solutions through involvement in patient associations and online forums. All the associations were built on the principle of “peer support,” which is based on mutual aid between people who suffer or have suffered from the same somatic or psychological illness or had the same life experience.

This experiential knowledge can be explored to understand the role of peers and associations in the appropriation of their cancer.

**Methods:**

A qualitative phenomenological study was undertaken through semi-structured interviews with 12 participants. Interviews were audio-recorded, transcribed verbatim, then analyzed by means of triangulation up to the point of theoretical saturation by a semio-pragmatic method.

**Results:**

Four categories emerged: (1) “Transforms a painful experience into a positive one. It mobilizes the human values of sharing, love, and humility, which facilitates resilience”; (2) “The characteristics of the association, a non-medical place between people sharing a common destiny, resonates with patients’ needs and improves their well-being”; (3) “The association transforms the patients’ experiences by facilitating engagement that leads to a patient-expert (empowerment)”; and (4) “Understanding what is happening to them is soothing, reassuring, because patients’ concerns need to be heard and their care understood”.

**Conclusions:**

This study highlights patient associations can serve as the mediator of NPI and facilate the empowerment of breast cancer patients.

**Practice implications:**

Educating health professionals in initial and continuing education about non-pharmacological interventions will be a major issue. Teaching the patient-centred approach to health professionals is one of the priorities in initial and continuing medical education.

## Background

In 2020, the number of new cancer cases was estimated at 20 490 862 worldwide up from 18.1 million in 2018 and 14.1 million in 2012 [1]. The number of cancer-related deaths was estimated at 10 021 864 in 2020 compared to 9.6 million in 2018 and 8.2 million in 2012. The World Health Organization (WHO) estimated that cancer had become the second leading cause of death responsible for more deaths than all coronary heart disease and stroke [1]. In 2020, breast cancer is the most frequently diagnosed cancer with 2 261 419 (11.7%) whereas in 2018, it was the second most frequently diagnosed cancer with 1.7 M cases. As of the end of 2020, there were 7.8 million women alive who were diagnosed with breast cancer in the past 5 years, making it the world’s most prevalent cancer [1].

Since the 2000s, cancer treatments have significantly improved, allowing either a cure or control of the disease. The standardized 5-year net survival (2005–2010) was estimated to be 87% over 10 years during the period between 1989 and 2010 [1]. Supportive care is used to reduce symptoms, extend autonomy, and improve quality of life. These methods include complementary therapies, non-pharmacological interventions (NPIs), and alternative and complementary medicine (CAM) [2]. Bruera and all defined the concept of supportive care in cancer as all the care and support needed by patients (physical, dietary, psychological and social) in addition to specific treatments (surgery, chemotherapy, radiotherapy) [3] NPI's can optimise radiotherapy in breast cancer by improving the physical and emotional well-being of patients [4]. The appropriation of cancer or resilience is an active process. Patients share their experience of the disease and use supportive care solutions through involvement in patient associations and online forums [5]. In a previous study [5], we analyzed online discussions on forums showing the interest of patients in NPI and the use of forums for support. With the help of health professionals to optimize person-centered support, forums support people’s informal learning on coping with a disease [6].

We were able to identify the common characteristics of the associations based on the promoters’ intentions [7]. A review of the literature shows that all the associations were built on the principle of "peer support", which is based on mutual help between people suffering or having suffered from the same somatic or psychological illness, or having lived the same life experience [8].

Associations also include person-centered supportive care to help people regain their lost existential balance and reappropriate identity values [7].

However, there is a lack of understanding of the role of patient organisations and peer support: How does the sharing of experience between peers in associations help them to live better with their chronic disease? By what process does peer support influence the patient's experience and appropriation of the disease? Finally, can patient organisations be considered as health interventions and be included in resilience strategies to help patients rebuild their lives? To answer these questions, a qualitative phenomenological approach focused on the lived experience of the actors seemed relevant. Because of the prevalence of breast cancer and the existence of several breast cancer patient associations in our area, we chose to interview patients who had contacted one of these associations. The aim of this study is to understand the role of peers and associations in the appropriation of their illness in patients with breast cancer by exploration of experiential knowledge.

## Methods

### Recruitment

The sample comprised of breast cancer patients belonging to a patient association. Three associations were contacted: Étincelle, Living Like Before, and InLife. A document explaining the purpose and modalities of the study was drafted. The interviews took place at either the association’s headquarters or the patients’ homes, except for one that was conducted over the telephone. The sampling followed the principle of a purposive sample [9], in which participants varied according to socio-professional level and membership in an association. The size of the sample was not established in advance, and data adequacy was determined from data emergence and saturation on material analysis.

### Data collection

Material for the analysis was gathered through in-depth interviews, using a guide drafted by two researchers, with phenomenologically oriented questions centered on the participant’s lived experiences (Table 1). The interviews took place between November 2017 and January 2019. Each of the 11 interviews lasted between 38 and 90 min. Participants ages 39 to 74 years freely described their personal lived experience without restraint. Table 2 summarizes the other characteristics guaranteeing a variation of patient experiences. Their responses were audio-recorded, then transcribed verbatim. The identifying features of all participants were anonymized after they signed a consent form for the analysis and publication of data. The researcher did not know the participants before interviewing them. Each verbatim transcript received a number to ensure anonymity. Ethical approval for this research was granted by a national ethic Committee for the Protection of Persons in accordance with the current regulation (IRB no IRB00010804). All procedures were performed in accordance with relevant declaration of Helsinki 's guidelines.

**Table 1:** Interview guide

1.Could you tell me about yourself? in general your history (person family work) Could you tell me about how you find yourself today more situated in time
2.Do you remember the day you were told about the disease, how it happened? What did you think, feel, do? How did you experience having this disease? What did it change in your daily life? How did you experience your treatments? Can you tell me about your treatment? (Chemotherapy, radiotherapy, surgery)? Apart from these treatments, have you been offered any other treatments (supportive care, NMIs, etc.)? Did this help you? Have you been offered a combination? 3. Have you ever participated in an association? What motivated you, why did you hear about it, why did you choose it, which association? What motivated you to join this association? Who told you about it? Why did you choose it? What does belonging to an association mean to you? How do you experience your association? Could you tell me about your participation, what you do? What do you think is your level of involvement? How has participating in an association changed the way you look at being ill? What has changed in your everyday life? your life and then your life as a patient? and how? relationship with others, identity, project?
4. What place do you think you have in the lives of other patients? Do you feel you have a particular expertise within this association? What place do you think you have in the lives of your peers?
5. If you were to advise one of your peers, what would you say?
6. Can you give me a picture (and/or draw me sth) that could represent you at these different times?

**Table 2:** Summary table of characteristics, age, membership of an association, occupation and duration of interviews with the patients questioned

Interview issue	Age	Association name	Occupation	Duration of the interview
S1	57 ans	Etincelle	Caregiver	53:15
S2	50 ans	Etincelle	Development manager	59:17
S3	70 ans	Etincelle	Teacher retired	50:28
S4	69 ans	Etincelle	Psychologist	57:57
S5	39 ans	Etincelle	Psychologist	51:58
S6	71 ans	Living as before	Tax officer	1:19:52
S7	74 ans	Living as before	Reporter then publisher	1:31:14
S8	47 ans	Etincelle /in life	Before accounting/ reviewer actually help at home	1:25:52
S 9	53 ans	The day after	Coach	37:58
S 10	48 ans		Patient association manadger	58:02
S11	59 ans	Ancient etincelle	Researcher inserm	1:30:00

### Phenomenological pragmatic analysis of the interview material

We used a phenomenological semio-pragmatic approach in accordance with our objective of exploring the lived experiences of female breast cancer patients. A semio-pragmatic analysis is a qualitative research method that proposes the logical ordering of empirical data according to their semiotic characterization, reducing the bias of interpretation without compromising the emergence of new results [9]. It is a specific phenomenological approach based on the category theory of CS Peirce [10], and a descriptive method for categorizing lived experience recorded from interview transcripts using the data ordering principle. Peirce demonstrated that only three categories were necessary and sufficient for describing any phenomenon. He called them “universal categories” or three “modes of being,” which are invariants of existence. In this method, the analyst takes into account all the semiotic elements of a text, including linguistic and contextual clues. They are linked by logical hierarchical semiotic relations. The concepts are at the top of the pyramid. They then presuppose the existing ones, which embody feelings. This formal principle of data ordering makes it possible to construct organized and relevant sets that will become first-level categories. Through a constant comparison process [11] with others verbatims, the analyst then enriches the emerging categories and their sub-categories or properties until the saturation point is reached and new categories are identified. Each category represents a dimension of the phenomenon being studied. The restitution of the meaning of the studied phenomenon is presented in the form of an integrative synthetic proposal taking into account the different dimensions. Using a formal principle limits the bias due to the researcher's interpretation and strengthens the validity of the results [12]. The steps of the method used are summarized in Table 3 [13].

**Table 3:** Steps of a pragmatic phenomenological analysis

Word by word transcription of recordings (verbatim)
A reading using a floating attention, followed by a focussed reading
Extracting signifying units from the text and grouping these units by themes
Collating textual and contextual meaningful semiotic elements and their semio-pragmatic characterisation
A first categorisation through a regrouping of these semiotic elements and of the signifying units in accordance with the research question
Enriching the categories by continuing comparison until theoretical saturation is reached
Placing the emerging categories in logical order and reducing them and their properties in order to model the ensemble in integrative semio-pragmatic statements

### Reliability criteria

The work is reported in conformity with the COREQ criteria [14]. The validity of coherence [15] has been respected by the congruence between the research objective and choice of methodological steps for the research, making them rigorously explicit. The triangulation of researchers was obtained between three experts in qualitative research who pooled their analyses. The three experts proceeded in two stages: each analysed the verbatims as they were transcribed, then they collated their results to compare the meaning of the emerging categories. A final meeting was held to agree on the formulation (statements) of these phenomenological categories. Three more interviews were performed once saturation of the material was reached.

Theoretical saturation is the result of the data analysis process as each interview is carried out. It is the continuing comparison procedure that allows the emerging categories to be enriched to the highest density. The density of a phenomenological category represents the level of information it contains about the phenomenon being studied. When the main categories are saturated, this means that there is no need for further interviews.

All participants consented to the release of the results of their interviews.

## Results

Four phenomenological statements emerged from the semio-pragmatic phenomenological analysis.

Four phenomenological statements emerged from the analysis. First, the association transforms a painful experience into a positive one to mobilize human values of sharing, love, and humility, which facilitates resilience. The association transforms the deleterious representation of the disease as a punishment that triggers feelings of guilt and anguish into something more positive. Feelings of total denial (“I was in total denial.” [S3]) were transformed to feelings of injustice (“No, it is not deserved, it is the opposite.” [S10], “Why am I sick when I have done everything?” [S8]). The patient may feel responsible or even guilty (“Being sick means feeling guilty and being punished.” [S3]). The illness can be a source of anguish (“I felt anguish when I learned I had cancer the second time.” [S3]). However, involvement in associations can also lead to a feeling of surprise, with cancer acting as the gateway to beautiful encounters. According to S3, “Having cancer is a negative experience, but joining an association allows cancer patients to meet with each other. I went from experiencing something very sad to having beautiful encounters that I would never have had without being sick. That is the good thing about joining associations.” Other responses reflecting the same sentiment are: “Offering my time to give back what I have received” (S1, S2); “The association has allowed me to rediscover my identity values, freedom of action and thought, commitment that gives me meaning, and restored confidence in my life” (S4, S10); “I am rediscovering my human values and now I really want to get involved” (S2); and “I need to have a sense of meaning in my life” (S4).

Second, the characteristics of an association, a non-medical organization for people sharing a common destiny, resonates with the needs of the patients and improves their with well-being.

According to S1, an association is defined as “a non-medical place for listening and understanding the person without judgement. It is a place where one has the freedom of expression, and everything is allowed without judgement, such as laughing and crying, as is the case in the medical environment. We do not talk about illness in these places; they are places of joy.” The association is a positive place where people belong to a community of destiny. It is “like a theater; we change, we reveal ourselves, we are without taboos and reluctance. I want to be useful” (S3). Joining an association means “to belong to a community” (S7) (S5), “the association helps them to advance in terms of their well-being as cancer patients” (S5). An association allows patients to give and receive from their peers: “I needed to find a place here. It is a cocoon where we do not talk about the disease, and people always have a smile on their faces” (S4); “I needed to have meaning in my life” (S4); and “I want to do something. It is also good to listen to patients and allow them to talk about their experiences” (S8).

Third, association transforms the patient’s experience by facilitating engagement that leads to one becoming a patient-expert (empowerment). Quoting S4, “I need to have meaning in my life. I am going to help and share my experience so I can be of some use, but it is not enough.” However, for S1, “the experience of the disease is singular, I do not want to speak for others, and I sometimes have the same experience or feeling of undesirable side effects, and I know what they can cost.” As for S2, she feels like, “a very modest expert in that respect. I can provide help, understanding, and support. It gives you more experience. Not all of us are equal: To be an expert, you have to have shared the same experience. Sometimes I cannot understand what others go through because my case is not the same. Others have experienced chemo, but I have not. But there are other things that I can share with them in terms of experience.” The response of S4 was: “I do not have to feel inferior in front of a doctor. He is skilled and I am someone who will discuss and ask questions, and even make suggestions… that we are on equal footing.”

Finally, literacy, and better understanding what is happening to them and their care through the involvement of peers and caregivers, enables them to find and understand information better. This develops their capacity and makes them feel peaceful and reassured. According to S1, “The small booklets were very well made and accessible to everyone. They did not use any difficult words They did not use any difficult words, allowing patients to better understand their treatment, “people need to understand their treatment (S11)”. S4’s response was, “Listen and take into account the patient’s words and allow us to understand them.” As for S8, “I was shocked to look up what it meant in the carcinologic dictionary. She did not tell me ‘You have breast cancer.’ It is a simple sentence. She gave me some papers and I looked up the meaning of what I had been told.”

## Discussion

This qualitative study provided a better understanding of the experience of cancer patients attending an association and addressed the objective to understand the role of peers and associations in the appropriation of brest cancer. Our four main emerging categories highlight the lived experience of cancer patients who feel the need to express themselves to caregivers and peers without the supervision of health professionals, using NPIs that allow them to experience well-being. They express the need to understand what is happening. These different factors enable them to change and take ownership of their disease, and even become expert patients. This study discuss these factors and show how they are intertwined.

Importance of the Patient Centred Approach as a medium for ownership of the disease: Peers involved in an association offer a person-centred approach which is essential in the helping relationship and the appropriation of their illness. The patient centeredness approach (PCA) is consistent with our four categories. It promotes change, resilience, and responsiveness. We owe the concept of PCA to M Stewart [16] who described this process along six dimensions, which he reduced to four. He describes the first as the fundamental component of the approach. It is the exploration of lived experience that can lead to a holistic biopsychosocial understanding of the person, which is the second dimension.

A sense of belonging to a Community of Destiny: Participants in forums [5] or associations are look:ing for a community of destiny, where people find a sense of belonging with others who have had the same experiences. People say it is not a way to exchange details about the disease and the suffering that comes with it, which they feared they would find there, but an outlet to discuss living with the disease, and how it affects their children. The patients likened this community spirit to [a sports team] with its competitive spirit of [winning together]. This is a concept developed by Maffesoli in [the time of tribes]; the association is like a tribe with its organization, customs, and habitus. Behind the [community of destiny],” there are concepts connoting sharing, mutual aid, [making oneself useful] to the other, and love. These concepts have emerged from the lived experiences of the patients in our study. The patients find that understanding what is happening to them is soothing and reassuring because their concerns need to be heard, which is in agreement with the literature [17].

Participating in a patient association improves health literacy: Health literacy (HL) highlights the importance of patients understanding their situation [17]. It is the degree to which one obtains, analyzes, and understands basic information [18]. Health understanding is a component of the implementation conditions and a pathway to patient engagement, commitment to adherence to interventional devices, and commitment to others in a helping relationship according to our findings. Health literacy is not simply a tool used to assess people’s understanding of an illness, but a “determinant of health” [19] where patients can play a more active role. It is an important predictor of successful self-management in cancer [20] and other chronic diseases [18]. Health literacy is also a determinant of therapeutic education; a low level of HL is a barrier to therapeutic education. Both approaches, HL and therapeutic education, have the same objective of increasing users' competencies [18, 19] for empowerment. People with low HL trust social media and blogs more than health professionals [21].

Patients can have the freedom to express themselves in associations to find their own solutions [22] without advice from health professionals, which constitutes support. For breast cancer, 72% of women use NPI's [23], but more than half do not mention them to their oncologist [24] for fear of disapproval or inability to help [25]. Moreover, alternative medicines can cause adverse effects, deleterious interactions with oncology treatments, and lost opportunities due to treatment delays [25].

Can the association be reclassified as a health intervention? If so, on what criteria [26, 27]? Is it a vector of NPI? Patients of the Etincelle association consider the association to be a health “intervention,” that delivers supportive care. For health professionals, the association may provide access to NPIs, but is not itself classified as one. The association can be seen as a social support device that allows the dispensation or exchange of emotional, instrumental, or information resources by non-professionals [28]. It responds to the important need for human equilibrium in social relations, with five essential functions: emotional support, social integration, the possibility of feeling useful and needed, confirmation of one’s value, and the acquisition of concrete and material help [28].

Our semi-pragmatic analysis allows a modelling.

“When the characteristics and design (conditions of implementation) of an association resonate with the components of the subjects’ lived experience in all its existential dimensions, this leads them to a process of adhesion, then of commitment, and finally of change, which gives them a feeling of newfound freedom.”

The process of change involves the transformation of the lived experience of the disease into a positive experience that goes hand in hand with a feeling of usefulness to others, well-being, and openness towards a new life project, taking the form of resilience.

From this perspective, the “association” itself takes the form of an NPI if we consider this definition (Ninot): [An NPI is a care encompassing more helpers with the aim of well-being, contrary to medicinal care, it is a complementary intervention with the aim of doing without medication]. Marion Carayol [29] hypothesized that the effects induced by the conditions of implementation of an intervention could be at the origin of the change, rather than the intervention itself. This hypothesis is confirmed by this qualitative research work. Other processes of change are described in the literature, resulting in a change of place, role, greater autonomy [30], and giving meaning to the experience [31]. Non-pharmacological interventions are described by our patients as “more supportive, encompassing care with the goal of wellness,” which is consistent with the literature [32]. Supervised adapted physical activity programs have been shown to benefit quality of life and prognosis in breast cancer patients as NPIs [33], and so has “mindfulness” [34].

Joining associations, sharing with peers, and NPIs were described by patients as a way of reclaiming their bodies. Listening to oneself and the caregivers, and sharing experiences with other patients is at the origin of a process of change that would allow patients to reappropriate their body in all its physical, social, and spiritual dimensions [35]. The association provides a time out from nursing one’s health. It is also a place of resilience. Most of the patients say they need to make themselves useful to others by helping them rebuild their lives. The patients’ life project is modified by the encounter with the disease [36], and this change in priority is reflected in our research. The patients refer to their past as either a facilitating element or, on the contrary, a source of difficulties. The person uses coping strategies [37]. The external resources surrounding the subject after the trauma of coping with chronic illnesses [38], and the return of self-confidence enable the subject to feel an improvement in both the physical and psychological quality of life, which is a reflection of our results. Patients also express the need to be accompanied and surrounded, a result which is found in the literature where the entourage contributes to the resilience of patients and the isolation being burdened with living with the disease [6, 39]. This is in accordance with our results.

These experiences have led to the emergence of so-called “expert” patients with different levels of expertise depending on their commitment and reflexivity. By moving away from the classic patient-disease-doctor triad, the emergence of the patient-expert changes hierarchical relationships and relationships to knowledge. It can also be a source of misunderstanding. According to the patients, “The association transforms the patient’s experience by facilitating engagement that leads to a patient-expert (empowerment). They live a statutory transition thanks to the association, which transforms the lived experience of the patients and guides them in the process of empowerment.” Glaser and Strauss [40] have described the properties of status transitions as “regular, planned and prescribed, but not necessarily always present. A transition can be desirable or undesirable” depending on the transition.

In the context of association as an “intervention,” there are multiple transitions: from patient to patient-witness, from isolated patient to patient-partner, from patient-in-doubt to committed patient, and from patient-partner to patient-expert. Not all patients go through these points of passage, but the association has a facilitating, mediating action to access them. The ultimate point of this dynamic process is empowerment, which refers to the mastery and control of the situation. What we can see is the transformation of an isolated, individual transition that gradually becomes a collective, with the sharing of the same pathway-destiny to benefiting the person within the patient association. This is an essential concept to apply in the search for change, as it is linked to the exploration of lived experience, which provides an opening towards the expectations and needs of the patient towards his or her life with the disease (i.e., towards the person, an approach advocated, for example, for people in migration situations) [41]. Change is promoted through the person-centered approach in accordance with our outcomes.

### Strengths and limitations

A phenomenological approach allows for a deep exploration of the lived experience, complementing epidemiological research. The validity of our results rests on the authenticity of the responses, and the triangulation of the analysis with the participation of two experts in the semio-pragmatic approach. This phenomenological approach is the only method that includes a formal ordering principle, which limits the interpretation bias of researchers in a quantitative analysis. This allows a better understanding of health behaviors and the mechanisms that lead to empowerment.

## Conclusion

This research work raises questions, but also highlights the contribution of the lived experience of patients treated for breast cancer on the understanding of their appropriation of the disease and the contribution of patient associations. When the characteristics and conception of the association resonate with the components of the subjects’ lived experience in all its existential dimensions, patients undergo a process of adhesion, then commitment, and finally of change, based on the various active ingredients and their induced effects emerging from the conditions of the implementation of the intervention. From this perspective, the patient association becomes a mediator of NPI, an inclusive and benevolent approach that makes it possible to direct each person towards relevant and personalized NPI.

### Practice implications

In the future, educating health professionals in initial and continuing education about NPIs will be a major issue. Currently, NPI and CAM approaches are often not well known in cancer care [42]. Teaching the patient-centered approach to health professionals is one of the priorities in initial and continuing medical education, as it is a major key to the professional patient-physician relationship, regardless of their beliefs about health and life. It allows us to ask patients about their use of NPIs, which would have a positive impact on the trust relationship [43–45].

The study shows that the place of patients and users in the French healthcare system is changing. They are becoming partners in their care. Teaching others about a disease based on one’s own experiences can introduce the patient-expert to the act of counselling or giving guidance to other patients, as was already the case in therapeutic education programs. There remains the question of patient associations. Changing the way we look at the role of the association invites us to reflect on the ethical consequences of this change. If the association is positioned as a care structure, then the problem of governance and status arises. Further research on the association should be carried out.

## Data Availability

The datasets used and/or analysed during the current study are available from the corresponding author on reasonable request.

## Abbreviations

NPI: Non-pharmacological interventions
CAM: Complementary medicine
PCA: Patient centeredness approach
HL: Health literacy
